# Ad26.COV2.S boosts antibody and T-cell responses following BNT162b2 vaccination

**DOI:** 10.1080/22221751.2021.2006581

**Published:** 2021-11-26

**Authors:** Sho Iketani, Lihong Liu, Manoj S. Nair, Abishek Chandrashekar, Hiroshi Mohri, Maple Wang, Dan H. Barouch, Yaoxing Huang, David D. Ho

**Affiliations:** a Aaron Diamond AIDS Research Center, Columbia University Vagelos College of Physicians and Surgeons, New York, NY, USA; b Department of Microbiology and Immunology, Columbia University Irving Medical Center, New York, NY, USA; c Center for Virology and Vaccine Research, Beth Israel Deaconess Medical Center, Harvard Medical School, Boston, MA, USA; d Ragon Institute of MGH, MIT, and Harvard, Cambridge, MA, USA; e Massachusetts Consortium on Pathogen Readiness, Boston, MA, USA; f Division of Infectious Diseases, Department of Internal Medicine, Columbia University Vagelos College of Physicians and Surgeons, New York, NY, USA

Multiple vaccines against SARS-CoV-2 (severe acute respiratory syndrome coronavirus 2) have been demonstrated to protect against COVID-19 (coronavirus disease 2019). While these vaccines have been promising in reducing symptomatic infections, the decay of neutralizing antibodies has been documented following vaccination [1]. Such decay is particularly pertinent due to the emergence of SARS-CoV-2 variants with relative resistance to vaccine-elicited antibodies [2, 3]. Consequently, losses in vaccine-mediated protection against select variants have been observed, and breakthrough infections in vaccinated individuals have been reported [4, 5, 6]. These studies suggest that a booster vaccine may be warranted. Although several studies have examined the effects of an additional homologous vaccination, the characterization of mixed vaccine regimens remains limited [7]. As heterologous vaccination series have many practical benefits, we investigated and report the cellular and antibody responses of seven healthy individuals who received a mixed regimen of two doses of BNT162b2 (Pfizer-BioNTech) followed by a third booster dose with the Ad26.COV2.S vaccine (Johnson & Johnson).

The seven individuals in this study were first confirmed not to have anti-nucleoprotein antibodies, suggesting that their responses reflected only the immunogenicity of the vaccines (Supplemental Figure 1A). All seven individuals had spike-binding antibodies at all timepoints tested, both against the non-variant strain and B.1.351, but had demonstrable loss in binding titre 4–6 months following their second vaccination with BNT162b2. Robust increases in binding titre were observed following a third vaccination with Ad26.COV2.S (Supplemental Figure 1B). These elicited antibodies were found to have neutralizing capability against all variant SARS-CoV-2 pseudoviruses, as well as all authentic SARS-CoV-2 strains tested except for the case of Vaccinee #4 against B.1.351 (Figure 1A and B). The increases in plasma neutralization titres (ID_50_) ranged from 9.4 to 17.6-fold in the pseudovirus neutralization assay and 12.2 to 23.3-fold in the authentic virus neutralization assay. Some individuals even had heightened neutralizing titre against SARS-CoV. For two of the vaccinees, we examined cellular immune responses, finding that such responses were also strongly bolstered by the third vaccination (Figure 1C).

In this study examining the cellular and antibody response of individuals receiving the Ad26.COV2.S vaccine following two doses of BNT162b2, a robust boost in the strength of neutralizing antibodies and cellular response, as well as breadth against SARS-CoV-2 variants was observed in all individuals. Although our cohort size is small, the similarity of results across individuals indicates a common effect. As observed in other studies, each of the individuals had a decay of neutralizing antibodies over time following BNT162b2 vaccination [1] and demonstrated reduced neutralizing titre against some SARS-CoV-2 variants [2]. This combination of temporal decay of antibody titre and the emergence of SARS-CoV-2 variants may therefore lead to a loss of protection in some individuals. We demonstrate herein that the Ad26.COV2.S vaccine administered as a third COVID-19 vaccine dose strongly boosts neutralizing antibody titres and cellular responses, including against rapidly spreading variants such as B.1.617.2 (delta variant) (Figure 1), reaching levels beyond that induced by the two BNT162b2 vaccinations alone. Importantly, this heterologous three-vaccine regimen mixing BNT162b2 and Ad26.COV2.S had similar trends as other reported homologous three-vaccine schedules and may therefore serve as one practical option for full control of this pandemic.

**Figure 1.**
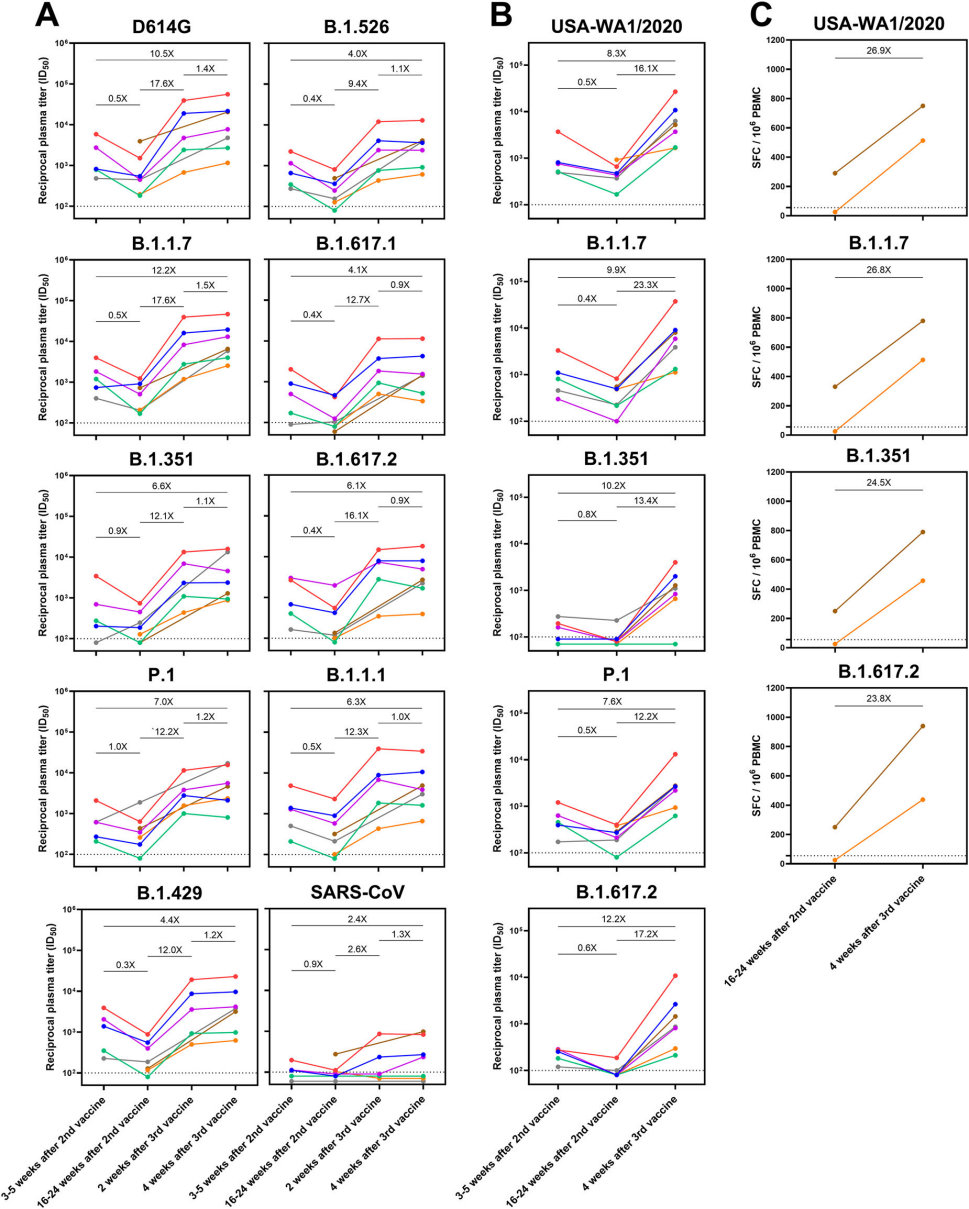
Immunogenicity of three SARS-CoV-2 vaccinations in healthy individuals. (A) Plasma samples were tested for neutralizing capability against recombinant vesicular stomatitis virus (rVSV) pseudotyped with spike from non-variant SARS-CoV-2 with D614G mutation, SARS-CoV-2 variants, or SARS-CoV. (B) Plasma samples were tested for neutralizing capability against authentic non-variant SARS-CoV-2 (USA-WA1/2020) and SARS-CoV-2 variants in a cytopathic effect reduction assay. (C) Peripheral blood mononuclear cells (PBMC) were tested for SARS-CoV-2 spike protein-specific cellular immune responses by IFNγ ELISPOT. In all panels, average fold change in reciprocal plasma titre (ID_50_) or spot forming cells (SFC) between two timepoints are denoted. The limit of detection (LOD) in both neutralization assays is ID_50_ = 100 and in the ELISPOT is 55 SFC/10^6^ PBMCs, and samples below the LOD are arbitrarily shown below the LOD to prevent overlapping datapoints. Colours denote individual vaccinees: blue = Vaccinee #1, red = Vaccinee #2, purple = Vaccinee #3, green = Vaccinee #4, orange = Vaccinee #5, brown = Vaccinee #6, grey = Vaccinee #7.

## Supplementary Material

20211101_Supplemental_Materials_for_EMI.docx
