# Supplementary material for: Ad26.COV2.S boosts antibody and T-cell responses following BNT162b2 vaccination
Source: Emerg Microbes Infect. 2021 Nov 26;10(1):2220–2. doi: 10.1080/22221751.2021.2006581 (PMC8635656; doi:10.1080/22221751.2021.2006581)
Supplement: 20211101_Supplemental_Materials_for_EMI.docx [file TEMI_A_2006581_SM1628.docx]

**Supplemental Figure 1.** **Binding profiles of individual samples against SARS-CoV-2 nucleoprotein (NP) and spike.** A, Two negative control plasma from healthy pre-SARS-CoV-2 patients and two positive control plasma from convalescent SARS-CoV-2 patients were included in the experiments. Data are shown as mean ± SEM of two technical replicates. Colors denote the samples from individual vaccinees at each timepoint: blue = Vaccinee #1, red = Vaccinee #2, purple = Vaccinee #3, green = Vaccinee #4, orange = Vaccinee #5, brown = Vaccinee #6, gray = Vaccinee #7. Negative control plasma are denoted in gold and positive control plasma are denoted in black. B, Plasma samples collected at the indicated timepoints were tested for binding to non-variant (WT) and B.1.351 SARS-CoV-2 spike. The average fold change in reciprocal plasma titer (EC_50_) between two timepoints are denoted. Colors denote individual vaccinees: blue = Vaccinee #1, red = Vaccinee #2, purple = Vaccinee #3, green = Vaccinee #4, orange = Vaccinee #5, brown = Vaccinee #6, gray = Vaccinee #7.

**
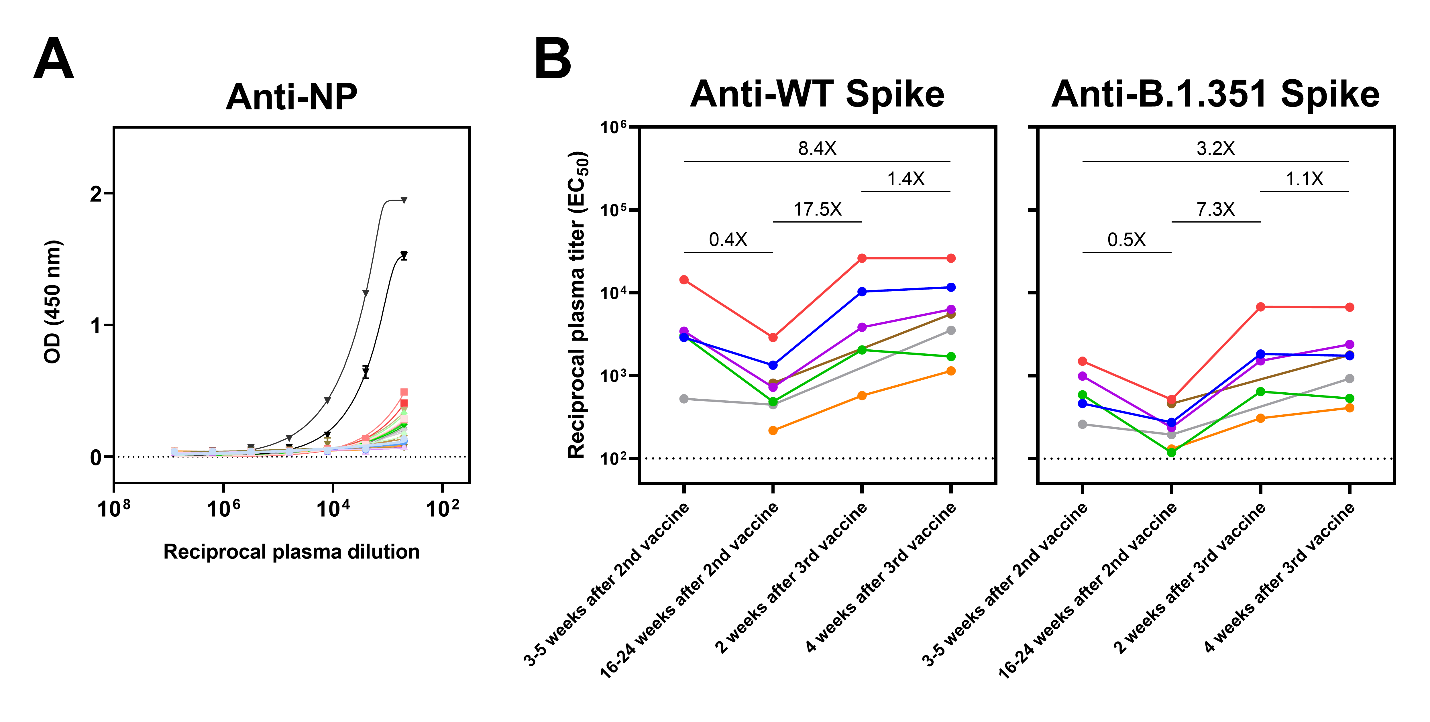
**

**Methods**

**Samples**

This study was reviewed and approved by the Institutional Review Board of Columbia University. The seven individuals in this study received their first and second doses of BNT162b2 vaccine three weeks apart, and then received a dose of Ad26.COV2.S vaccine as a third vaccination four to six months after the second vaccination. Plasma and PBMCs were collected three to five weeks after the second vaccination, 16 to 24 weeks after the second vaccination, two weeks after the third vaccination, and four weeks after the third vaccination.

**Quantification of antibody responses**

Enzyme-linked immunosorbent assay (ELISA) to quantify anti-SARS-CoV-2 spike and nucleoprotein antibodies, recombinant vesicular stomatitis virus-based SARS-CoV-2 pseudovirus neutralization assay, and authentic SARS-CoV-2 neutralization assay, were performed as previously described [1].

**Quantification of cellular responses by IFNγ ELISPOT**

Enzyme-linked immunosorbent spot (ELISPOT) assay was conducted as previously described [2]. ELISPOT plates were coated with mouse anti-human IFNγ monoclonal antibody from MabTech at 1 μg per well and incubated overnight at 4 °C. Plates were washed with DPBS, and blocked with R10 medium (RPMI with 10% heat-inactivated FBS with 1% of 100× penicillin–streptomycin, 1 M HEPES, 100 mM sodium pyruvate, 200 mM l-glutamine, and 0.1% of 55 mM 2-mercaptoethanol) for 2–4 h at 37 °C. SARS-CoV-2 pooled spike peptides from WA1/2020, B.1.1.7, B.1.351, and B.1.617.2 (21st Century Biochemicals) were prepared and plated at a concentration of 2 μg per well, and 100,000 cells per well were added to the plate. The peptides and cells were incubated for 15–20 h at 37 °C. All steps after this incubation were performed at room temperature. The plates were washed with ELISPOT wash buffer and incubated for 2–4 h with biotinylated mouse anti-human IFNγ monoclonal antibody from MabTech (1 μg ml^−1^). The plates were washed a second time and incubated for 2–3 h with conjugated Goat anti-biotin AP from Rockland (1.33 μg ml^−1^). The final wash was followed by the addition of Nitor-blue Tetrazolium Chloride/5-bromo-4-chloro 3 ‘indolyphosphate p-toludine salt (NBT/BCIP chromagen) substrate solution for 7 min. The chromagen was discarded and the plates were washed with water and dried in a dim place for 24 h. Plates were scanned and counted on a Cellular Technologies Limited Immunospot Analyzer.

**References**

1. Liu L, Wang P, Nair MS, et al. Potent neutralizing antibodies against multiple epitopes on SARS-CoV-2 spike. Nature. 2020 Aug;584(7821):450-456.

2. Chandrashekar A, Liu J, Yu J, et al. Prior infection with SARS-CoV-2 WA1/2020 partially protects rhesus macaques against re-infection with B.1.1.7 and B.1.351 variants. Sci Transl Med. 2021 Sep 21:eabj2641.
